# CPDMS: a database system for crop physiological disorder management

**DOI:** 10.1093/database/baaf031

**Published:** 2025-04-22

**Authors:** Jae-Hyeon Oh, Hwang-Weon Jeong, Il Pyung Ahn, Seon-Hwa Bae, Sung Mi Kim, Eunhee Kim, Su Jung Ra, Jinjeong Lee, Hye Yeon Choi, Young-Joo Seol

**Affiliations:** National institute of Agricultural Sciences, Rural Development Administration, 370, Jeonju-si, Jeollabuk-do 54874, Republic of Korea; National institute of Agricultural Sciences, Rural Development Administration, 370, Jeonju-si, Jeollabuk-do 54874, Republic of Korea; National institute of Agricultural Sciences, Rural Development Administration, 370, Jeonju-si, Jeollabuk-do 54874, Republic of Korea; National Institute of Horticultural and Herbal Science, Rural Development Administration, 100, Wanju-gun, Jeollabuk-do 55365, Republic of Korea; National institute of Agricultural Sciences, Rural Development Administration, 370, Jeonju-si, Jeollabuk-do 54874, Republic of Korea; National institute of Agricultural Sciences, Rural Development Administration, 370, Jeonju-si, Jeollabuk-do 54874, Republic of Korea; National institute of Agricultural Sciences, Rural Development Administration, 370, Jeonju-si, Jeollabuk-do 54874, Republic of Korea; National institute of Agricultural Sciences, Rural Development Administration, 370, Jeonju-si, Jeollabuk-do 54874, Republic of Korea; Phyzen Genomics Institute, 605, Baekgung Plaza 1st Building, 13, Seongnam-daero 331-gil, Bundang-gu, Seongnam-si, Gyeonggi-do 13558, Republic of Korea; Technology Cooperation Bureau, Rural Development Administration, 300, Jeonju-si, Jeollabuk-do 54874, Republic of Korea

## Abstract

As the importance of precision agriculture grows, scalable and efficient methods for real-time data collection and analysis have become essential. In this study, we developed a system to collect real-time crop images, focusing on physiological disorders in tomatoes. This system systematically collects crop images and related data, with the potential to evolve into a valuable tool for researchers and agricultural practitioners. A total of 58 479 images were produced under stress conditions, including bacterial wilt (BW), *Tomato Yellow Leaf Curl Virus* (TYLCV), *Tomato Spotted Wilt Virus* (TSWV), drought, and salinity, across seven tomato varieties. The images include front views at 0 degrees, 120 degrees, 240 degrees, and top views and petiole images. Of these, 43 894 images were suitable for labeling. Based on this, 24 000 images were used for AI model training, and 13 037 images for model testing. By training a deep learning model, we achieved a mean Average Precision (mAP) of 0.46 and a recall rate of 0.60. Additionally, we discussed data augmentation and hyperparameter tuning strategies to improve AI model performance and explored the potential for generalizing the system across various agricultural environments. The database constructed in this study will serve as a crucial resource for the future development of agricultural AI.

**Database URL**: https://crops.phyzen.com/

## Introduction

The agricultural sector is undergoing a transformation driven by the integration of digital technologies, particularly artificial intelligence (AI), machine learning, and big data analytics [1]. As global food demand continues to rise, there is an increasing need for precise and efficient crop management methods that can enhance productivity while minimizing resource use [2]. Traditional crop disease diagnosis methods have primarily relied on manual inspections and expert knowledge, which are time-consuming and prone to human error and variability [3]. These limitations are particularly problematic in large-scale agricultural operations, where timely and accurate disease detection is critical to maintaining crop health and maximizing yield [4].

Recent advancements in AI, particularly deep learning, have demonstrated the potential for automating plant disease diagnosis through image analysis [5]. For instance, Mohanty *et al*. (2016) [6] demonstrated that deep learning models can achieve expert-level accuracy in identifying plant diseases from images. However, the success of these AI models heavily depends on the availability of large annotated datasets that include diverse crop types, disease conditions, and environmental variables [7]. Smartphones equipped with high-resolution cameras and internet connectivity offer an unparalleled opportunity to collect vast datasets directly from agricultural fields. These devices can be essential tools for real-time data collection and systematic management in the field. When combined with robust database systems and sophisticated AI algorithms, real-time data collection via smartphones can significantly enhance the scalability and accessibility of precision agriculture.

This study presents the development of a smartphone-based crop image collection, annotation, and management system, focusing on physiological disorders such as BW, TYLCV, TSWV, drought, and salinity in tomatoes. This system integrates advanced database management with AI-based analysis to support both research and practical applications. By building a large-scale annotated image database, this study aims to improve crop management practices and contribute to the advancement of digital agriculture.

However, several challenges remain in applying AI to agriculture. One of the major issues is the variability of environmental conditions, which can significantly affect the appearance of plant diseases and the performance of AI models trained in controlled environments [8]. To address these challenges, this study focuses on developing AI-based diagnostic tools that leverage diverse and extensive datasets, ensuring adaptability across various agricultural environments. The ultimate goal of this research is to provide a scalable and practical solution for improving crop disease diagnosis, contributing to enhanced agricultural productivity and sustainability.

## Materials and methods

### Plant materials and pathogen/stress treatments

The tomato varieties used in the study were Hawaii7996, Seogwang, AVTO0101, Super Doterang, Tori, Lucky, and Ten-Ten, making a total of seven varieties. These varieties were subjected to infection by BW, TYLCV, and TSWV. For BW infection, the *Ralstonia solanacearum* strain (18 644, WR-1) obtained from the Korean Agricultural Culture Collection (KACC) of the Rural Development Administration was used to inoculate the tomato plants. TYLCV and TSWV infections were induced using mechanical inoculation with *Agrobacterium tumefaciens*, and the infected plants were maintained under controlled laboratory conditions (Table 1).

**Table 1.** Information on tomato disease symptoms

Name of the disease	Pathogen nucleic acid type	Pathogen	Specific amplification region for each pathogen	Primer information
Bacterial Wilt	DNA	*Ralstonia solanacearum* Species Complex (RSSC)	lpxC(RSc2836 or RS06240)	F: 5ʹ-ATGTCGAGCGGTAGCATC-3’
			intergenic region upstream of RSc2836	R: 5ʹ-TGCGATCGTCGATCGATC-3ʹ * Product size (bp) : 150
TYLCV disease		TYLCV	V1 protein gene	F: 5ʹ-CGATCGATCGATCGATCG-3’
			TYLCV coat protein gene region	R: 5ʹ-GCTAGCTAGCTAGCTAGC-3ʹ * Product size (bp) : 200
TSWV disease	RNA	TSWV	NSm	F: 5ʹ-CAGCAACACGGTCAAGACAA-3’
			The movement protein gene, involved in the movement of TSWV	R: 5ʹ-TGGTCCAGGTGTTGATGATG-3ʹ * Product size (bp) : 120

### Plant growth and management practices

Plants were grown in 15 cm diameter pots filled with a mixture of peat and perlite in a 3:1 ratio. Each cultivar was represented by 15 plants (three biological replicates, five plants per replicate), and all plants were maintained under controlled environmental conditions: temperature was maintained at 25°C ± 2°C, relative humidity at 60% ± 5%, and light intensity at 200 µmol/m^2^/s with a 16-h/8-h light/dark cycle. A balanced NPK fertilizer was applied weekly to ensure optimal growth conditions. Experiments were conducted in a controlled environment chamber with consistent ventilation and CO_2_ levels.

### Disease inoculation and image acquisition

For disease inoculation, plants were infected at the four-leaf stage. Images were acquired 7 days post-inoculation to capture disease progression. For drought and salinity treatments, plants were subjected to stress at the six-leaf stage. Drought stress was induced by water deprivation, while salinity stress was applied using a 200 mM NaCl solution. Both treatments lasted for 14 days, and images were acquired at the end of the treatment period.

In addition, drought stress (water deprivation) and salinity stress (200 mM NaCl treatment) were applied to the seven tomato varieties, starting from 28 days after germination. The resulting stress responses were captured as image data.

### Data collection and annotation

**Figure 1.**
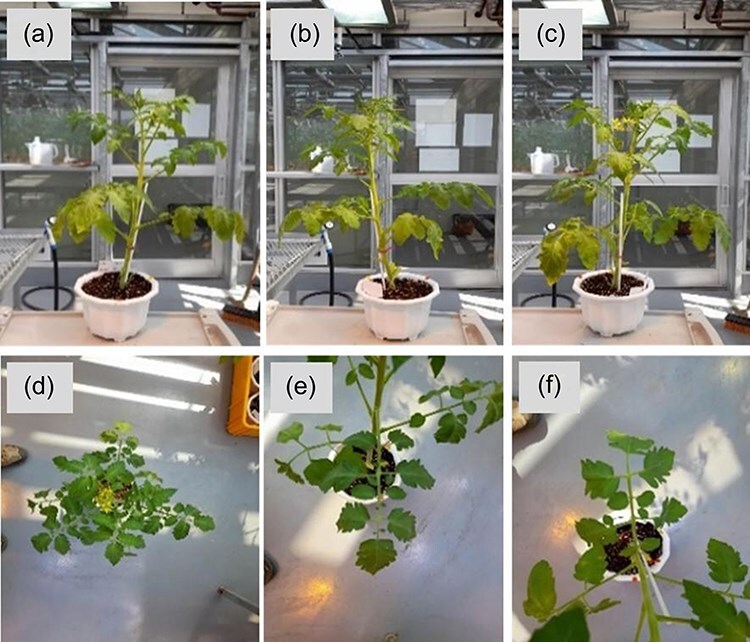
Collection of healthy and diseased plant images from various angles. (a). Front view image (0°), (b) front view image (120°), (c) front view image (240°), (d) top view image, (e) petiole image (upper leaf), (f) petiole image (lower leaf).

Images of each tomato variety were collected at various growth stages and positions (front views at 0°, 120°, 240°, top view, and petiole views of upper and lower leaves) under conditions of BW, TYLCV, and TSWV infection, as well as environmental stress (salinity and drought) (Fig. 1). The collected images were annotated with information about the specific plant parts affected by physiological disorders and the severity of the symptoms. The annotations were performed manually using the LabelImg tool (https://github.com/tzutalin/labelImg), an open-source image annotation tool. Each image was annotated for disease symptoms, stress types, and plant growth stages. The annotated data were stored in a relational database for systematic management. This high-quality dataset served as the foundation for AI model training.

### System development

**Figure 2.**
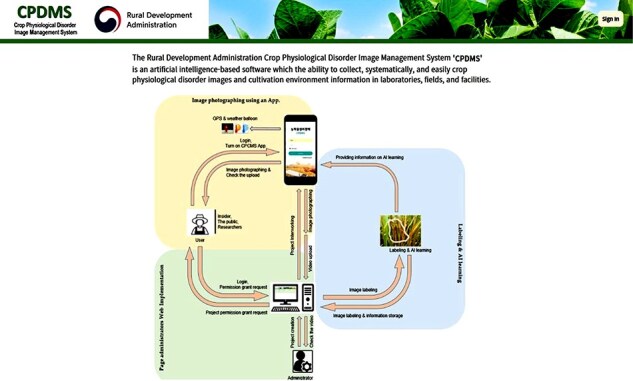
A snapshot of the CPDMS web database. The website is organized into an introduction and a physiological disorder collecting flow chart.

The system developed in this study is a web-based platform that provides a responsive web application optimized for both Android and Apple devices. This system is designed to allow users to access it through a mobile web browser without the need to install a separate app. Instead of using React Native [9], HTML5 [10], and CSS3 [11] were employed to maximize compatibility and responsiveness across various browser environments. The system integrates data collection and database management functions to ensure efficient operation, with the collected data being securely stored in a central database in real time (Fig. 2). Currently, the annotation process is handled using an external tool, with future plans to fully integrate this feature into the system.

### Database design and integration

To efficiently manage the large volume of collected image data, a relational database structure was adopted. This database is managed on an on-premises server, providing stable access and regular updates, allowing users to quickly search and access data based on specific conditions. The database is designed with compatibility for deep learning frameworks such as TensorFlow [12] and PyTorch [13], enabling annotated datasets to be exported in formats suitable for use in these frameworks.

### AI model training and validation

To diagnose tomato diseases, the YOLOv5 [14] framework was used to train a deep learning model. This framework was chosen for its efficiency in real-time object detection. The dataset was divided into training (70%), validation (20%), and testing (10%) sets, with a total of 24 000 images used for training, 6857 for validation, and 13 037 for testing. The images were collected under various environmental conditions, which refer to different stress conditions (biotic and abiotic) applied within the same controlled laboratory environment. For example, drought, salinity stress, and pathogen infection (BW, TYLCV, and TSWV) were considered different environmental conditions. The model’s performance was evaluated using metrics such as precision, recall, and mean Average Precision (mAP).

To enhance the model’s performance, data augmentation techniques (e.g. random rotation, zoom, and lighting adjustments) were applied, and hyperparameter tuning was conducted to find the optimal model configuration. In the future, the model will be expanded to generalize across various environments and crops, with plans for additional data collection and model retraining.

### Pathogen quantification using qRT-PCR

Quantitative reverse transcription polymerase chain reaction (qRT-PCR) was used to quantitatively assess pathogen proliferation. Infected tissues were collected from susceptible varieties (AVTO0101, Hawaii7996, Seogwang) and resistant varieties (Lucky, Tori) infected with Bacterial Wilt (BW), followed by RNA extraction and conversion to cDNA for qRT-PCR analysis. Each experiment was performed with three biological replicates, and samples were collected from five plants per replicate. The primer sequences used for qRT-PCR are listed in Table 1. This method enabled the analysis of the correlation between visible disease symptoms and actual pathogen proliferation, allowing for a quantitative evaluation of disease progression. The same method was applied to plants infected with TYLCV, where RNA was extracted and viral proliferation was quantified through qRT-PCR.

## Results

### Field performance of the web application

**Figure 3.**
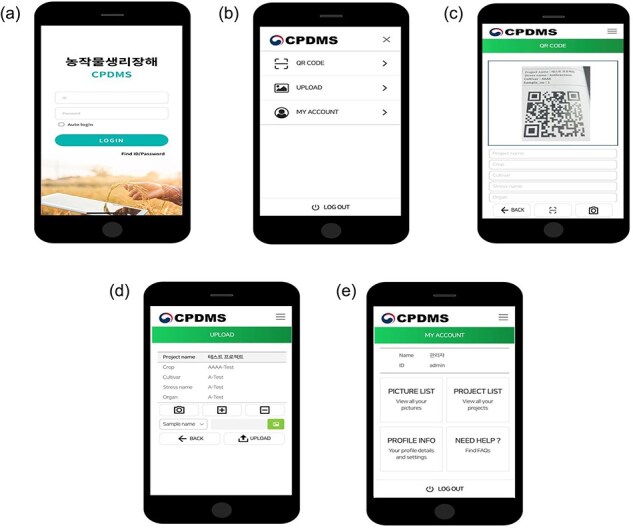
Application of physiological disorder collecting images using a smartphone. (a) Application main screen snap. (b) Major functions of application. (c) QR code-based management of object meta-information. (D) Upload of image. (E) My account includes user information and collected information such as image.

The developed web-based application was tested under various environmental conditions, allowing users to download images and annotate them in the web environment. The application provides a user-friendly interface and responsive performance, enabling agricultural workers to collect data in real time. Laboratory tests demonstrated that the system performed reliably across different stress conditions and crop growth stages within a controlled environment, allowing users to consistently capture disease symptoms (Fig. 3).

### Image collection and annotation

**Figure 4.**
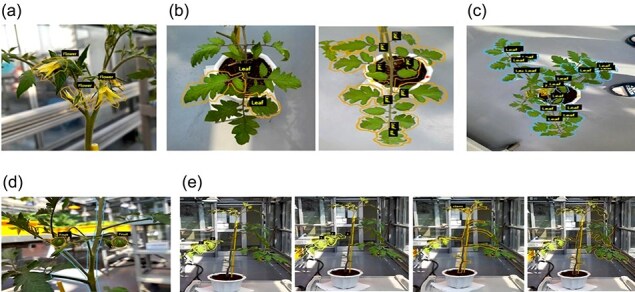
Plant disease symptom image annotation. (a) FW(Flower), (b) LF(Leaf), (c) TOP(Top view), (d) FR(Fruit), and (e) SV(Side view).

Each variety of tomato was observed at various growth stages following infection with BW, TYLCV, and TSWV, with 10 732 images, 22 343 images, and 3027 images collected, respectively. Additionally, each tomato variety was exposed to salinity and drought stress, with a total of 22 377 images collected at various growth stages (Table 2). Detailed annotations of the collected images enabled the identification of affected plant parts and the severity of physiological disorders (Fig. 4). The systematic organization of these data in a relational database facilitated efficient training of AI models, as detailed in Supplementary Fig. S1.

**Table 2.** Number of images collected for each tomato variety under different conditions

Disease symptom	BW	TYLCV	TSWV	Physiological disorder	Total
Image yield	10 732	22 343	3027	22 377	58 479

### Database functionality and accessibility

**Figure 5.**
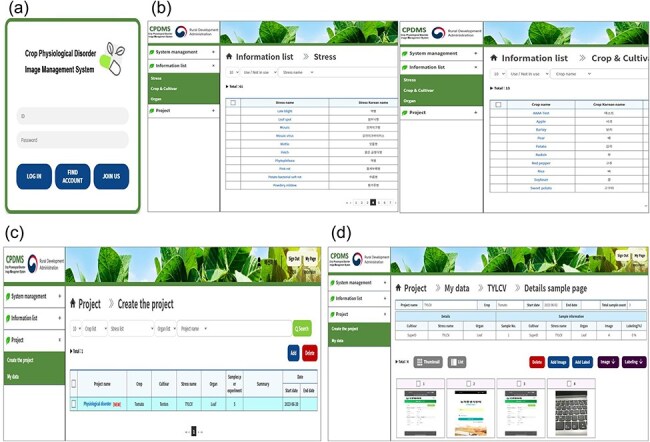
Physiological disorder image database system. (a) Main screen interface of crop physiological disorder image management system (b) Crop stress and crop information included in the informational list category (c) Project creation list. (d) The database can be aligned in a list form of images taken by the smartphone.

The relational database efficiently manages large-scale image data and is designed to allow users to quickly filter and access specific data (Fig. 5). The database is compatible with TensorFlow and PyTorch, enabling the export of annotated datasets for AI model training. The current system is operated on an on-premises server, supporting stable data access and regular updates, which enhances research productivity.

### AI model accuracy and utility

**Figure 6.**
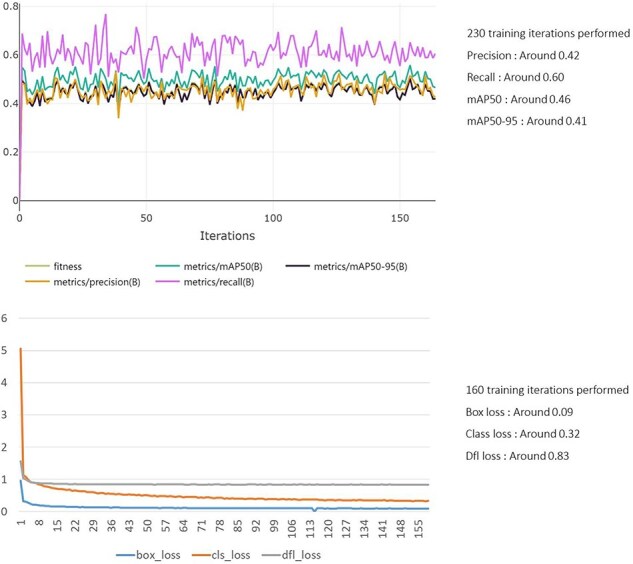
Artificial intelligence training process.

To develop the AI training model, the YOLOv5 framework was employed to detect and classify physiological disorders in tomatoes. Data folders were organized, and training parameters were configured before initiating the model training process. The trained model achieved a mAP of 0.46 and a recall of 0.60 on the test dataset (Fig. 6), demonstrating reliable detection accuracy across various disease conditions (Supplementary Fig S2). The model’s performance was assessed across different stages of TYLCV, TSWV, and BW (Bacterial Wilt). For BW, early stages were challenging to distinguish from healthy plants due to similar phenotypic characteristics, resulting in lower recognition accuracy. However, for TYLCV and TSWV, recognition rates improved as the diseases progressed, achieving an overall accuracy exceeding 95% (Supplementary Fig S2). Additionally, the model maintained consistent performance under common stress conditions, such as drought and salinity, highlighting its robustness across diverse agricultural environments.

### Pathogen quantification using qRT-PCR

**Figure 7.**
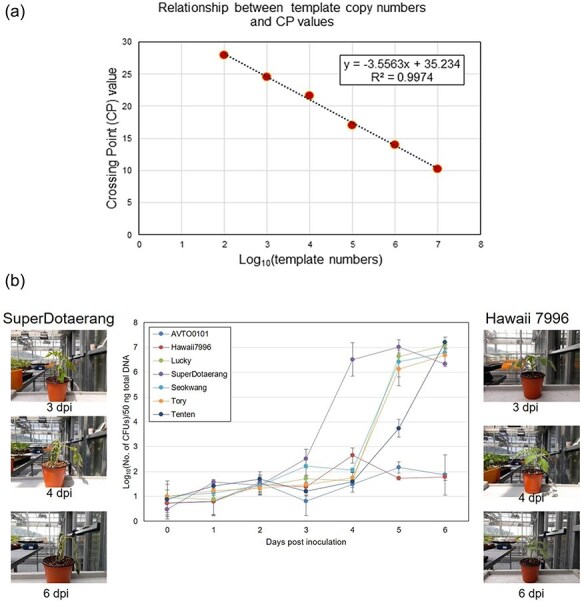
Correlation between *Ralstonia solanacearum* cell count and CP value, and pathogen content in resistant and susceptible tomato tissues. (a) Correlation between the number of *R. solanacearum* (Bacterial Wilt pathogen) cells and CP value. (b) Measurement of Bacterial Wilt pathogen content in infected tissues of resistant and susceptible tomato plants.

qRT-PCR analysis revealed significant differences in pathogen proliferation between susceptible and resistant varieties. In susceptible varieties infected with BW, pathogen proliferation increased rapidly, with Hawaii7996 showing the lowest proliferation rate, indicating high resistance to the pathogen. In contrast, pathogen proliferation was limited in resistant varieties (Fig. 7). In the TYLCV and TSWV infection experiments, viral proliferation was active in susceptible varieties, whereas it was suppressed in resistant varieties (Supplementary Fig S3 and S4). These results clearly demonstrate the correlation between visible disease symptoms and actual pathogen proliferation.

## Discussion

This study developed a smartphone-based crop image collection and management system, providing an efficient and scalable solution for real-time data collection and management in agricultural fields. The system is designed to rapidly diagnose major diseases, including physiological disorders in tomatoes, across various environments, and store the data in a central database for future analysis and research. Notably, through the web-based platform, users could easily access the system via mobile web browsers without installing an application, and the data were securely stored in real-time in the central database. This approach significantly enhanced user accessibility, increasing the system’s practicality and boosting the efficiency of field operations.

The deep learning model trained using the YOLOv5 framework produced reliable results under various disease conditions, with particularly high accuracy for specific diseases. However, the relatively low mAP suggests that further improvements are needed through additional data augmentation and hyperparameter tuning. Future research aims to enhance the model’s generalization performance by collecting data from more diverse environments and crops, ultimately achieving higher accuracy and recall rates.

While significant progress has been made, several challenges persist in applying AI to agriculture. One critical issue is the variability of environmental conditions, which can influence AI model performance and complicate accurate disease diagnosis. For instance, symptoms of the same disease may vary depending on environmental factors, necessitating model training strategies that account for such variables. This study demonstrates that collecting data from diverse environments and incorporating these variables into model training significantly improves diagnostic accuracy. Furthermore, ensuring user accessibility, enhancing data security, and addressing the long-term sustainability of AI systems are essential to facilitate practical adoption in the field. The development of intuitive user interfaces and system stability was also identified as key factors for improving user experience and operational efficiency.

The system developed in this study focuses on specific crops (tomatoes) and diseases (physiological disorders), and its applicability to other crops and diseases may be limited. Therefore, future research should aim to develop an expanded system that includes data from a variety of crops and diseases. Additionally, advanced techniques such as transfer learning or ensemble learning could be applied to further improve AI model performance. In conclusion, this study aims to bridge the gap between cutting-edge AI technology and practical agricultural applications by providing a scalable, efficient, and accessible crop management solution. Through the development of a comprehensive smartphone-based system, this study seeks to enhance the accuracy and effectiveness of crop disease diagnosis, ultimately contributing to improved agricultural productivity and sustainability. This study has laid an important foundation for the advancement of precision agriculture, opening up the potential for better agricultural management solutions through the integration of AI and digital technologies. With continuous research and system improvements, this system has the potential to become a powerful tool for enhancing agricultural productivity and promoting sustainable farming practices.

## Conclusions

The smartphone-based crop image collection and management system developed in this study is a web-based platform accessible through a mobile web browser, providing optimized displays for both Android and Apple devices. This system allows agricultural workers to efficiently collect high-quality crop images in real time from the field, with the collected data securely stored and managed in a central database. Additionally, the system integrates advanced database management with AI-based analysis functions to support both research and practical applications, with plans to incorporate AI analysis functions in the future. Laboratory tests have demonstrated that the system performs reliably and consistently across diverse stress conditions, and users highly value its intuitive interface and dependable performance. In particular, deep learning-based AI analysis using the YOLOv5 model has produced reliable results in detecting and classifying physiological disorders in tomatoes. This system is poised to become a powerful tool for advancing digital agriculture and supporting sustainable farming practices, with scalability for application to various crops and environments in the future.

## Supplementary Material

baaf031_Supp

## Data Availability

All data used in this study are publicly available at https://crops.phyzen.com/ and https://crops.phyzen.com/app
