# Supplementary material for: CPDMS: a database system for crop physiological disorder management
Source: Database (Oxford). 2025 Apr 22;2025:baaf031. doi: 10.1093/database/baaf031 (PMC12013473; doi:10.1093/database/baaf031)
Supplement: baaf031_Supp [file baaf031_supp.zip › suppl_data/Supplementary File.docx]

**Article type:** Original Article

**Running title:** Real-Time Crop Disorder Data Collection System

**Title:**

CPDMS: A database system for crop physiological disorder management

**Authors:**

Jae-Hyeon Oh^1^, Hwang-Weon Jeong^1^, Il Pyung Ahn^1^, Seon-Hwa Bae^1^, Sung Mi Kim^1^, Eunhee Kim^1^, Su Jung Ra^1^, Jinjeong Lee^1^, Hye Yeon Choi^2^, Young-Joo Seol^3,^*

**Author Affiliations:**

^1^Gene Engineering Division, National institute of Agricultural Sciences, Rural Development Administration, 370, Jeonju-si, Jeollabuk-do 54874, Republic of Korea.

^2^Phyzen Genomics Institute, Seongnam-si, Gyeonggi-do 13558, Republic of Korea

^3^Rural Development Administration, 300, Jeonju-si, Jeollabuk-do 54874, Republic of Korea

***Corresponding author:** Young-Joo Seol, Tel: +82-63-238-4656; Fax: +82-63-238-4654, jhoh8288@korea.kr

# Supplementary Information

## Supplemental Figures


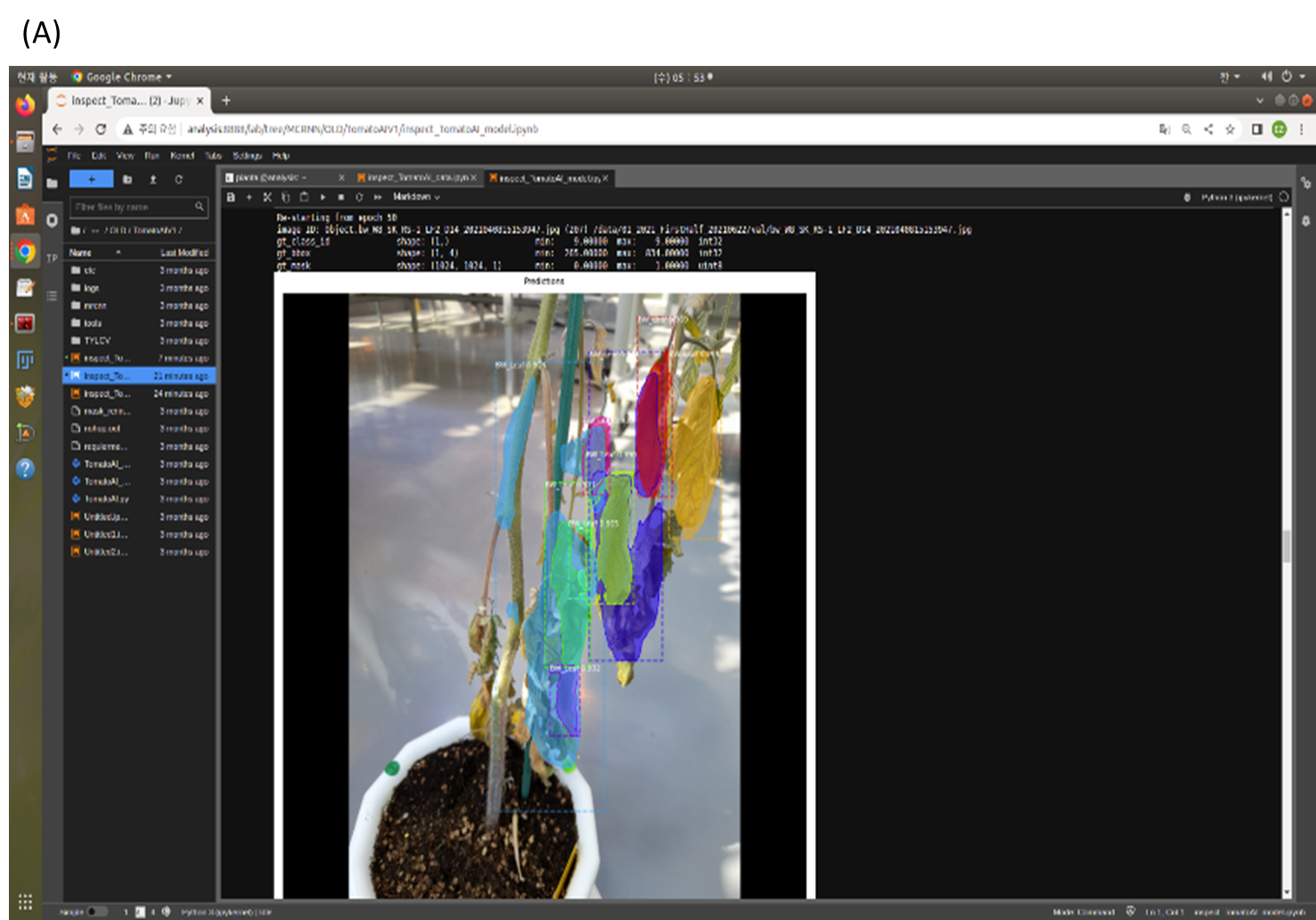


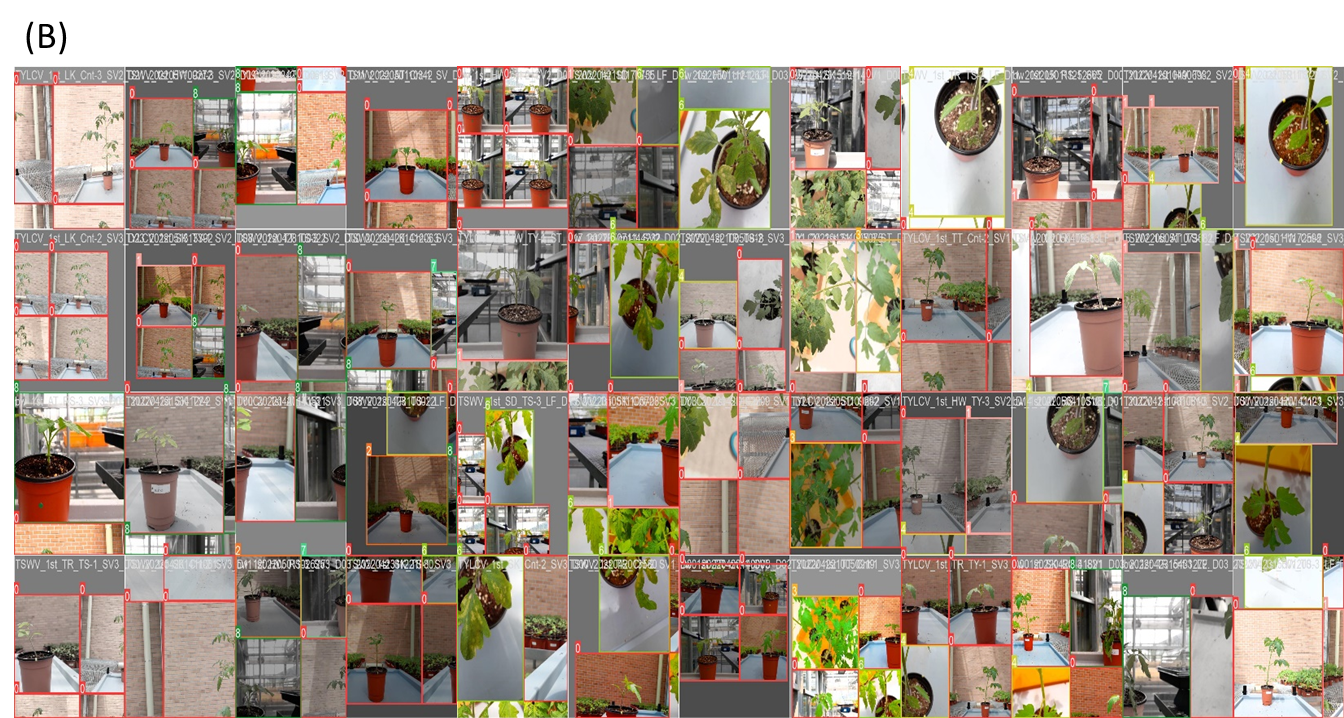


**Fig S1**. Development of a Pipeline and Interface for AI Training.

(A) Interface development using JupyterLab: A management module, data format conversion function for training sets, and data augmentation functionality were implemented within JupyterLab to evaluate AI models produced through this platform.

(B) AI training data augmentation: Data augmentation was performed on the training data by applying operations such as mosaic, color transformation, resizing, and horizontal flipping.


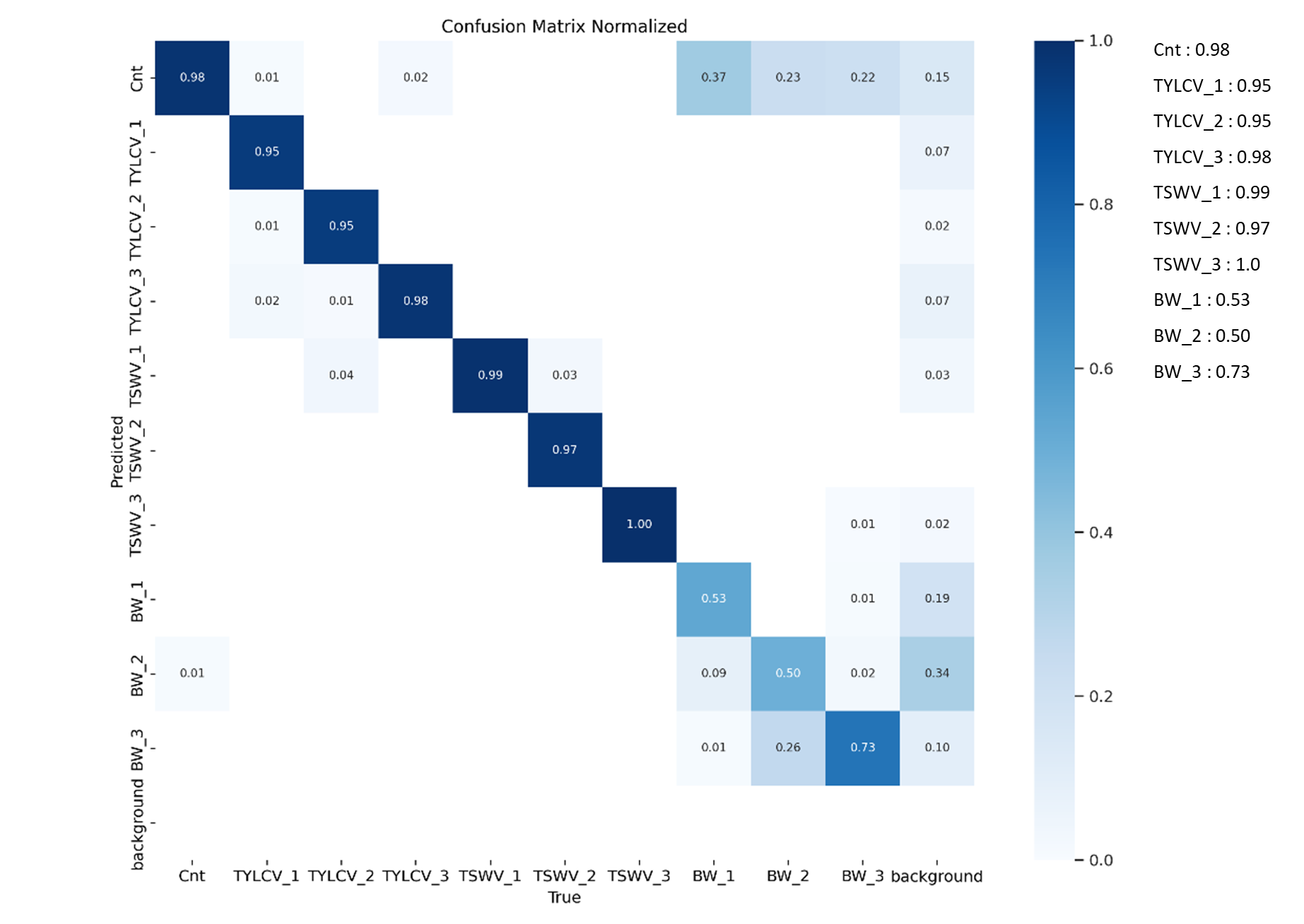


**Fig S2.** Accuracy of AI Training Results (Confusion Matrix)

The model was trained in three stages, and the results were as follows: healthy plants achieved an accuracy of 98%, TYLCV showed a gradual increase in recognition accuracy, ranging from 95% to 98% depending on the stage, and TSWV achieved 97% to 100% accuracy. In the case of Bacterial Wilt, the accuracy was 53% in the early stage and 73% in the later stage. The lower accuracy for Bacterial Wilt is likely due to the similarity between the symptoms of wilting caused by the disease and those caused by drought stress.


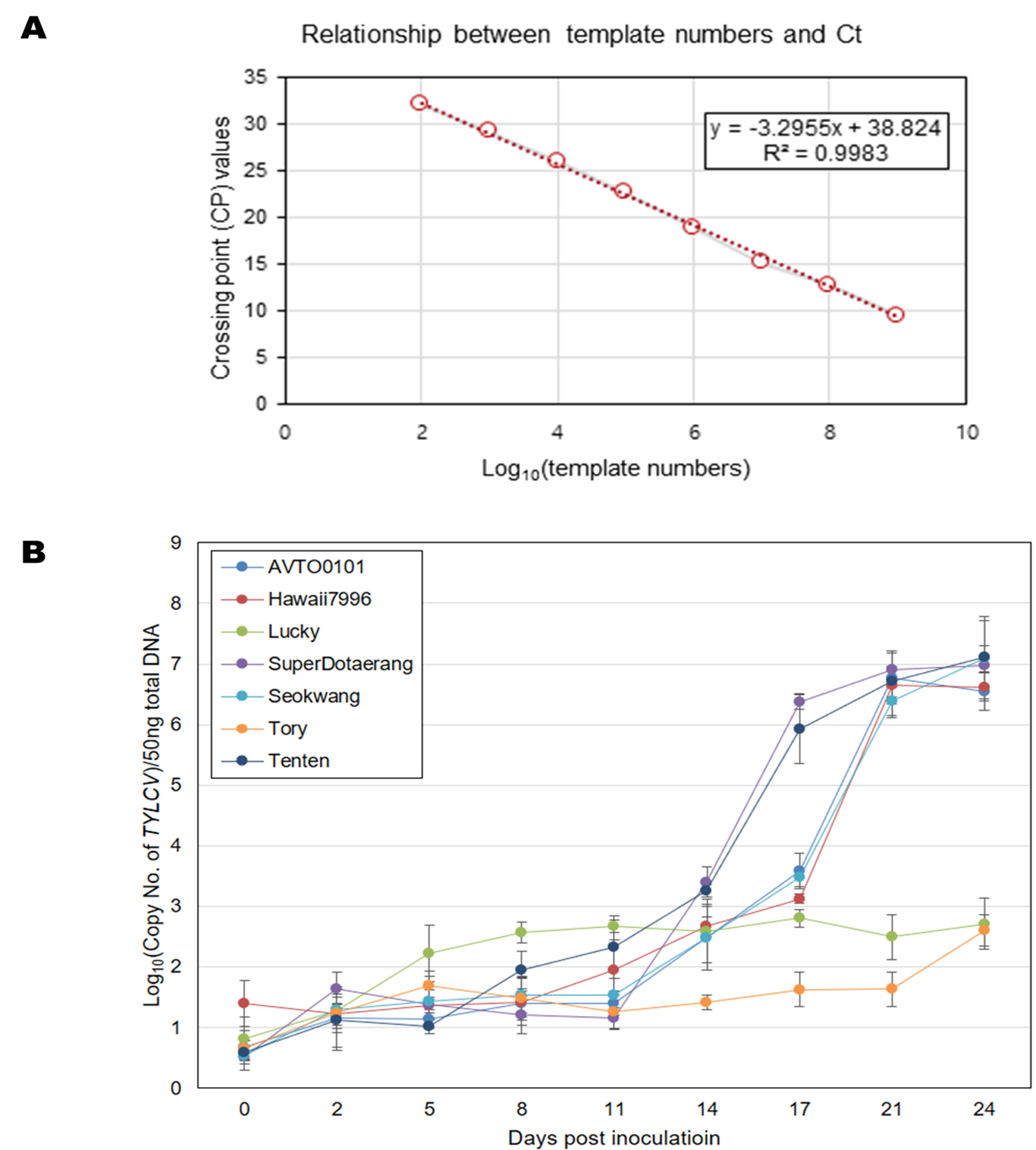


**Fig S3.** Correlation Between TYLCV Template Count and CP Value, and Pathogen Content in Resistant and Susceptible Tomato Tissues. (A) Correlation between the number of TYLCV templates and CP value. (B) Measurement of virus pathogen content in infected tissues of resistant and susceptible tomato plants to *Tomato Yellow Leaf Curl Virus* (TYLCV).


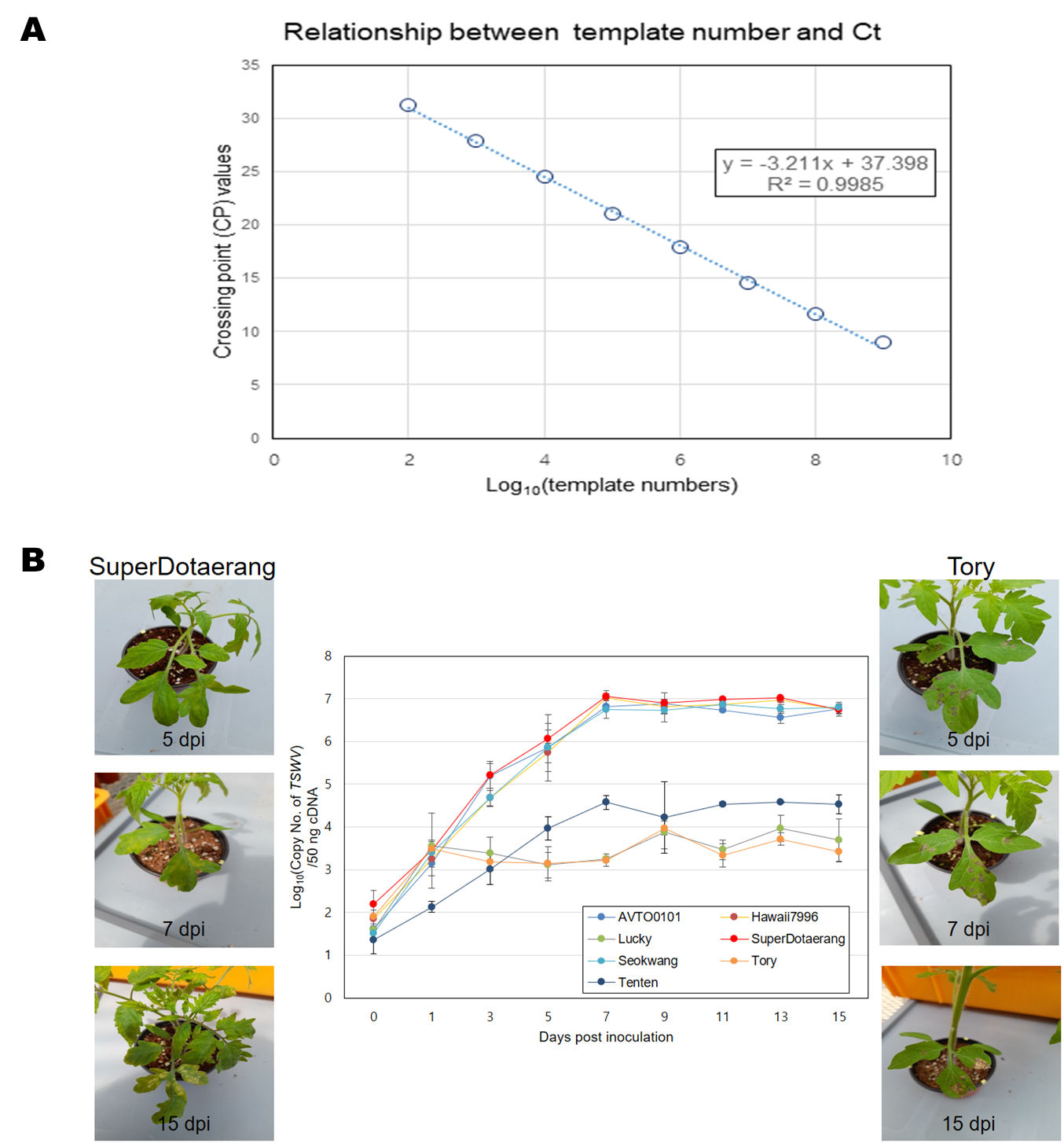


**Fig S4.** Correlation Between TSWV Template Count and CP Value, and Pathogen Content in Resistant and Susceptible Tomato Tissues. (A) Correlation between the number of TSWV templates and CP value. (B) Measurement of virus pathogen content in infected tissues of resistant and susceptible tomato plants to *Tomato Spotted Wilt Virus* (TSWV).
